# The Proliferative Response to p27 Down-Regulation in Estrogen Plus Progestin Hormonal Therapy is Lost in Breast Tumors

**DOI:** 10.1016/j.tranon.2018.02.011

**Published:** 2018-03-07

**Authors:** Mark D. Aupperlee, Anastasia Kariagina, Nicole Zaremba, Marc D. Basson, Richard C. Schwartz, Sandra Z. Haslam

**Affiliations:** * Breast Cancer and the Environment Research Program, Michigan State University, East Lansing, MI; † Department of Physiology, Michigan State University, East Lansing, MI; ‡ Department of Microbiology and Molecular Genetics, Michigan State University, East Lansing, MI; § Department of Surgery, University of Southern California, Los Angeles, CA; ¶ Department of Surgery, University of North Dakota, Grand Forks, ND

## Abstract

Increased proliferation and breast cancer risk has been observed in postmenopausal women receiving estrogen (E) + progestin hormone replacement therapy (HRT). Progestin action is mediated through two progesterone receptor (PR) isoforms, PRA and PRB, with unique transcriptional activity and function. The current study examines hormonal regulation of PR isoforms in the normal postmenopausal human breast and the mechanism by which progestins increase proliferation and breast cancer risk. Archival benign breast biopsies from postmenopausal and premenopausal women, and luminal breast tumor biopsies from postmenopausal women, were analyzed for regulation of PRA and PRB expression by E and E+medroxyprogesterone acetate (MPA). In the postmenopausal breast without HRT, PRA and PRB expression was decreased compared to the premenopausal breast. Both E (n = 12) and E+MPA (n = 13) HRT in the postmenopausal breast were associated with increased PRA and PRB expression, increased nuclear cyclin E expression, and decreased nuclear p27 expression compared to no HRT (n = 16). With E+MPA HRT, there was a further decrease in nuclear p27 and increased Receptor Activator of NF-kappa B Ligand (RANKL) expression compared to E-alone HRT. In luminal breast cancers, E+MPA HRT (n = 6) was also associated with decreased nuclear expression of the cell cycle inhibitor p27 compared to E HRT (n = 6), but was not associated with increased proliferation. These results suggest that p27 mediates progestin-induced proliferation in the normal human breast and that regulation of this proliferative response by E+MPA is lost in breast tumors.

## Introduction

Progesterone (P) and synthetic progestins have been implicated in the etiology and progression of breast cancer in both animal models and the human breast [1]. In the human postmenopausal breast, hormone replacement therapy (HRT) with the conjugated equine estrogen (E) + the progestin medroxyprogesterone acetate (MPA) increases breast cancer risk over E alone [2], [3], [4], [5], [6]. Following the Women’s Health Initiative findings on E+MPA HRT in 2002, a decline in HRT use was associated with decreased breast cancer incidence [7], [8]. However, in both the Women’s Health Initiative randomized trial [9] and the E3N cohort [10], a significantly elevated risk of breast cancer continued, even after stopping HRT.

The progesterone receptor (PR) mediates the action of P and synthetic progestins in the mammary gland (reviewed in [11]) and exists as two isoforms, PRA and PRB. The full-length isoform PRB, and the truncated isoform PRA, are encoded from the same gene and mRNAs. Ligand-activated PRs dimerize (A:A, B:B, and A:B) and localize to the nucleus where they repress or activate PR-target genes. *In vitro* studies using human breast cancer cell lines have shown that PRA and PRB have unique transcriptional activity and function [12]. Thus, relative expression of PRA and PRB is an important determinant of progestin action in the human breast. In normal premenopausal breast epithelium, PRA and PRB are co-expressed at similar levels and altered PR isoform (i.e., PRA) expression has been observed in the progression of breast cancer [13], [14], [15], [16]. Alterations in PR isoform expression may be due to transcriptional regulation or may also be due to increased turnover of active ligand-bound PRB [17]. Total PR rather than PR isoform expression is usually measured in the clinical context, and while it is well-established that E regulates overall PR expression [18], hormonal regulation of individual PR isoforms PRA and PRB *in vivo* in the human breast has not been studied.

E+MPA HRT in normal breast is associated with increased proliferation, increased epithelial content, and increased breast density [19]. In addition, E+MPA HRT has been linked to increased risk of more aggressive breast cancers that are associated with a higher rate of breast cancer death [20]. Proliferation of breast epithelium is highest during the luteal phase of the menstrual cycle when endogenous P level is highest [19], [21], [22], [23], [24]. Treatment of cultured primary normal human breast cells with P increased proliferation through activation of pathways involved in DNA replication licensing [25]. While it is clear that progestins in the postmenopausal breast influence proliferation and breast cancer risk, the detailed mechanism of progestin action *in vivo* in the normal, intact human breast remains poorly understood.

In this study, the effect of HRT on expression of PRA, PRB and potential downstream regulators of progestin action was examined in normal postmenopausal breast tissue samples and in luminal breast tumors from postmenopausal women who had received hormonal therapy with either E alone or E+MPA.

## Materials and Methods

### Normal Premenopausal and Postmenopausal Human Breast Samples

Archival human breast samples were used from a cross-sectional, observational study carried out to study breast tissue from cycling, premenopausal (n = 10) and postmenopausal (n = 31) women undergoing surgical breast biopsy at Lansing, Michigan area hospitals. Of the postmenopausal women, 25% of those with no HRT experienced a surgical menopause; 75% of those with E HRT; 7.7% of those with E+MPA HRT. In the original tissue collection, biopsies were carried out to diagnose suspicious palpable lesions upon physical exam or suspicious mammographic densities. The protocol for fresh tissue collection of samples was approved by the University Committee on Research Involving Human Subjects and Institutional Research Review Boards of the participating hospitals; written informed consent was obtained from each patient. Biopsies collected for study were kept on ice, then fixed in 3.7% buffered formalin within 2 h of surgery for paraffin embedding.

Profiles of the study populations of postmenopausal women are summarized in Table 1. Postmenopausal women were defined as those who had experienced 12 consecutive months of amenorrhea, had a bilateral oopharectomy at least 1 year before biopsy, or were 55 years of age or older. Subjects were placed into one of three categories: (1) no HRT, defined as not having taken hormones for 1 year before surgery; (2) E-alone; or (3) E+progestin. HRT subjects were defined as those taking hormones for at least 3 months continuously up to the day of surgery. All hormones were taken on a continuous, daily basis. Subjects had taken E in one of two forms: conjugated equine estrogens (dose, 0.3–2.5 mg; n = 11) or micronized estradiol (dose, 0.5 mg; n = 1). The progestin taken was medroxyprogesterone acetate (MPA; dose, 2.5–5 mg; n = 13), which was taken in combination with conjugated equine estrogens (n = 11) or micronized estradiol (n = 2). Herein, all types of estrogens are referred to as E. The specific progestin used in all studies was MPA, so all E+progestin HRT is referred to as E+MPA HRT.

**Table 1:** Postmenopausal Breast Sample Subject Characteristics

Characteristic	No HRT	E	E+MPA
Number of subjects	n = 16	n = 12	n = 13
Mean age (y) (range)	63.7 ± 12.0 (41-77)	63.9 ± 9.7 (43-79)	62.1 ± 9.7 (54-88)
Body mass index (kg/m2)	27.7 ± 5.1	27.3 ± 6.8	27.5 ± 6.8
Time on HRT (y) (range)	n/a	15.8 ± 8.9 (4-34)	7.4 ± 4.4 (3-20)
Menopausal status (%)			
Natural	75.0%	25.0%	92.3%
Surgically induced	25.0%	75.0%	7.7%
Reproductive History			
Nulliparous (%)	12.5%	8.3%	0%
Parous (%)	87.5%	91.7%	100%
Mean no. pregnancies (range)	3.9 ± 2.4 (2-7)	2.9 ± 1.3 (1-5)	3.2 ± 1.3 (2-7)
Mean no. deliveries (range)	2.7 ± 1.6 (1-6)	2.8 ± 1.3 (1-4)	2.9 ± 1.2 (2-6)
Age at 1st delivery (y) (range)	22.2 ± 4.0 (16-31)	20.4 ± 2.9 (16-26)	22.1 ± 3.4 (17-28)

Premenopausal subjects were divided into two categories depending on the phase of the menstrual cycle: (1) follicular, days 1–14; or (2) luteal, days 15–28.

### Postmenopausal Human Breast Cancer Samples

Archival formalin-fixed paraffin-embedded blocks of human breast cancer samples were obtained from Sparrow Hospital in Lansing, Michigan. These samples were unrelated to the samples of normal breast tissue examined in this study. The protocol for obtaining archival samples and identifying relevant samples was approved by the Human Research Protection Program at Michigan State University and the Institutional Research Review Board of Sparrow Hospital. Medical record analysis was performed at Sparrow Hospital to select samples originating from postmenopausal women receiving E HRT (n = 6) or E+MPA HRT (n = 7). Information was not available on whether any of the tumor samples were derived from women that had surgically induced menopause. After selection of samples to examine, de-identified archival breast tumor samples (Table 2) were analyzed at Michigan State University. Pathology reports on all the tumor samples chosen indicated that they were ER and PR positive, suggesting a luminal subtype for all breast tumors. Breast tumors from women that had not received HRT were not available for this study.

**Table 2:** Postmenopausal Breast Tumor Sample Subject Characteristics

E	Age (y)	Time on HRT (y)	Lobular vs Ductal	Invasive vs In Situ	ER	PR	HER2
1	66	10	Ductal	Invasive	Positive	Positive	Weak Positive
2	61	16	Ductal	Invasive	Positive	Positive	Negative
3	66	13	Ductal	Invasive	Positive	Positive	Weak Positive
4	48	9	Ductal	Invasive	Positive	Positive	Positive
5	46	2	Ductal & Lobular	Invasive	N/A	N/A	N/A
6	80	N/A	Ductal & Lobular	Invasive	Positive	Positive	N/A
Mean	61.2 +/− 5.2	10 +/− 5.2					
E+MPA							
1	54	1	Lobular	Invasive	Positive	Positive	N/A
2	58	N/A	Lobular	Invasive	Positive	Positive	Negative
3	51	20	Ductal	Invasive	Positive	Positive	N/A
4	52	N/A	Ductal	Invasive	Positive	Positive	N/A
5	67	N/A	Lobular	Invasive	Positive	Positive	N/A
6	58	8	Metastatic	Invasive	Positive	Positive	Negative
Mean	56.7 +/− 5.9	9.7 +/− 9.6					

### Immunofluorescence

Sections (5 μm) were mounted onto 3-aminopropyl-triethoxysilane (Sigma, St. Louis, MO)-coated cover slips, and assayed by immunofluorescence on nonserial sections. The number of tissue samples assayed varied because, in some cases, there was not enough tissue for all assays, or some tissue sections only contained lobules or ducts, and not both. Sections were deparaffinized, rehydrated, and subjected to antigen retrieval (20 min at 121°C, 16 p.s.i. in citrate buffer (pH 6.0)) prior to immunofluorescent detection.

Single antibody immunofluorescence labeling was performed with rabbit polyclonal anti-cyclin E (1:50, Santa Cruz, sc-481), anti-p27 (1:250, Santa Cruz, sc-528), anti-RANKL (1:500, Novus), anti-Amphiregulin (Areg) (1:100, Neomarkers, Ab-1 RB-257-P1), and mouse monoclonal anti-Ki67 (1:100, Dako, MIB1) antibodies. Primary antibodies were recognized by appropriate secondary antibodies conjugated to Alexa 488 (Molecular Probes, Invitrogen). Sections were counterstained with 4′,6-diamidino-2-phenylindole (DAPI) (Invitrogen).

Detection of PR isoforms PRA and PRB in the human breast has been performed using mouse monoclonal antibodies specific to PRA and to PRB [15], [26]. To increase the efficiency of PRB detection and prevent possible cross-reactivity between anti-mouse secondary antibodies used to detect the mouse monoclonal PRA primary antibody, a new rabbit polyclonal anti-human PRB antibody was developed against amino acids 56-73 of human PRB. The specificity of this new rabbit polyclonal anti-PRB antibody (G1699) was confirmed using T47D cells expressing PRA only (T47D-YA), expressing PRB only (T47D-YB), or without PR expression (T47D-Y) (Supplemental Figure 1). Specific, robust detection of PRB by the G1699 antibody was demonstrated by immunofluorescence (Supplemental Figure 1*A*) and by Western blot (Supplemental Figure 1*B*) in T47D-YB and T47Y cells. Specific detection of PRA by hPRa7 with immunofluorescence was also confirmed by immunofluorescence in T47D-YA cells (Supplemental Figure 1*A*). As previously reported [26], hPRa7 detects only PRA by immunofluorescence, but detects both PRA and PRB under the denaturing conditions of immunoblot (Supplemental Figure 1*B*). The differential specificity of hPRa7 between immunofluorescence in intact cells and immunoblot suggests that the availability of the recognized epitope is dependent on protein folding and that this epitope is masked in native PRB [26]. Thus, the efficacy of specific detection of PRA may be dependent upon conditions of fixation and antigen retrieval. Detection of PRB by immunofluorescence with G1699 antibody co-localized with the established mouse monoclonal anti-PRB antibody, hPRa6 [26] (Supplemental Figure 1*C*).

Double immunofluorescence labeling was performed with antibodies directed to PRA and PRB, PRB and p63, or PRB and ERα. PRA was detected with a mouse monoclonal anti-PRA antibody (hPRa7, Neomarkers). PRB was detected with a rabbit polyclonal anti-PRB antibody (G1699) generated against amino acids 56-73 of human PRB. P63 was detected with a mouse monoclonal anti-p63 antibody (Clone 4A4, Thermo Scientific). ERα was detected with a mouse monoclonal anti- ERα antibody (Clone 6F11, Leica Biosystems).

### Statistical Analysis

Results are shown as mean ± standard error of the mean (SEM). For postmenopausal human breast samples, a minimum of 5 samples were analyzed from the no HRT, E HRT, and E+MPA HRT groups. For premenopausal human breast samples, 4 samples were analyzed for luteal and 3 samples for follicular phases of the menstrual cycle. For human luminal breast tumors, 6 samples from women receiving E HRT and 6 samples from women receiving E+MPA HRT were analyzed. A minimum of 1000 cells were analyzed from each sample. Differences were considered significant at *P* < .05 using Student’s *t* test.

## Results

### Normal Postmenopausal Breast Sample Population Profile Similarities

Subjects in the three postmenopausal groups were similar for age, body mass index, and reproductive history, but there were some differences among the groups (Table 1). 75% of the E-alone HRT subjects had experienced a surgical menopause and their mean time on HRT was twice as long as that of women taking E+MPA HRT (15.8 vs. 7.4 y). The majority of women receiving no HRT or E+MPA HRT experienced natural menopause.

### PRA and PRB Expression and Regulation in the Normal Breast

**Figure 1:**
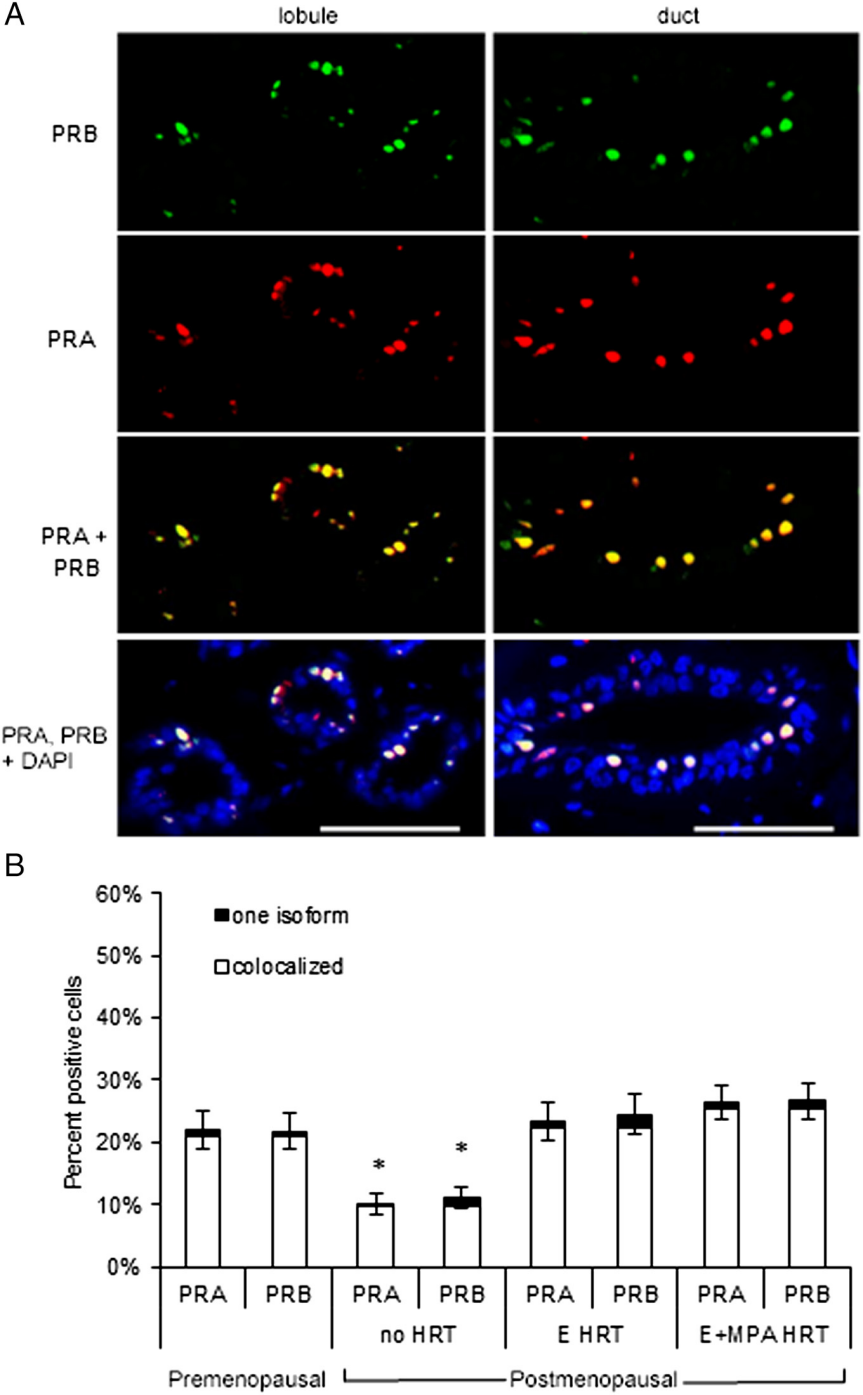
The effect of the hormone replacement therapy on PRA and PRB expression in the postmenopausal breast. (A) Immunofluorescent detection of PRA (red), PRB (green), and PRA+PRB (yellow) in ducts and lobules of normal postmenopausal breast of women receiving no HRT, E HRT, or E+MPA HRT. Nuclei were stained with DAPI (blue). Scale bar = 25 μm. (B) The percentage of PRA and PRB positive cells was decreased in the postmenopausal breast with no HRT (*P < .05).

PRA and PRB regulation and co-localization in the postmenopausal and premenopausal breast were examined by dual immunofluorescence (Figure 1*A*). PRA and PRB expression were maintained in the breast epithelium of postmenopausal women who received no HRT, but decreased 2-fold compared to premenopausal women (Figure 1*B*). PRA and PRB expression were increased to a similar level in the postmenopausal breast by HRT with E alone (2.2 fold) or E+MPA (2.4 fold). Across all postmenopausal treatment groups and in the premenopausal breast, over 90% of PR positive cells co-expressed PRA and PRB. A small, consistent percentage of epithelial cells in the premenopausal breast expressed only PRA (1.0%) or only PRB (0.8%). The postmenopausal breast also contained a small percentage of epithelial cells expressing only PRA (0.5%-1.1%) or only PRB (1.3%-2.2%).

E, acting through estrogen receptor α (ERα), regulates expression of PR [18]. We confirmed that PRB expression co-localized with ERα across all postmenopausal breast samples, regardless of HRT treatment (Supplemental Figure 2*A*). Since PRA and PRB co-localized, it is likely that most hormone receptor positive cells in the postmenopausal breast co-express ERα, PRA, and PRB. We also confirmed that PRB was primarily detected in p63-negative luminal epithelial cells (Supplemental Figure 2*B*).

### RANKL and p27 Associate with Estrogen + MPA-Induced Proliferation

Given previous findings that E and E+MPA HRT increased epithelial proliferation in the postmenopausal breast, with the greatest increase in proliferation with E+MPA [19], we sought to identify potential downstream mediators of E and E+MPA HRT-induced proliferation. This was carried out in the same archival postmenopausal breast samples as Hofseth et al [19], where we measured proliferation by Ki67 detection. In the current study, we focused on targets regulated by progestin to explain the observed increase in proliferation with the addition of MPA to HRT.

**Figure 2:**
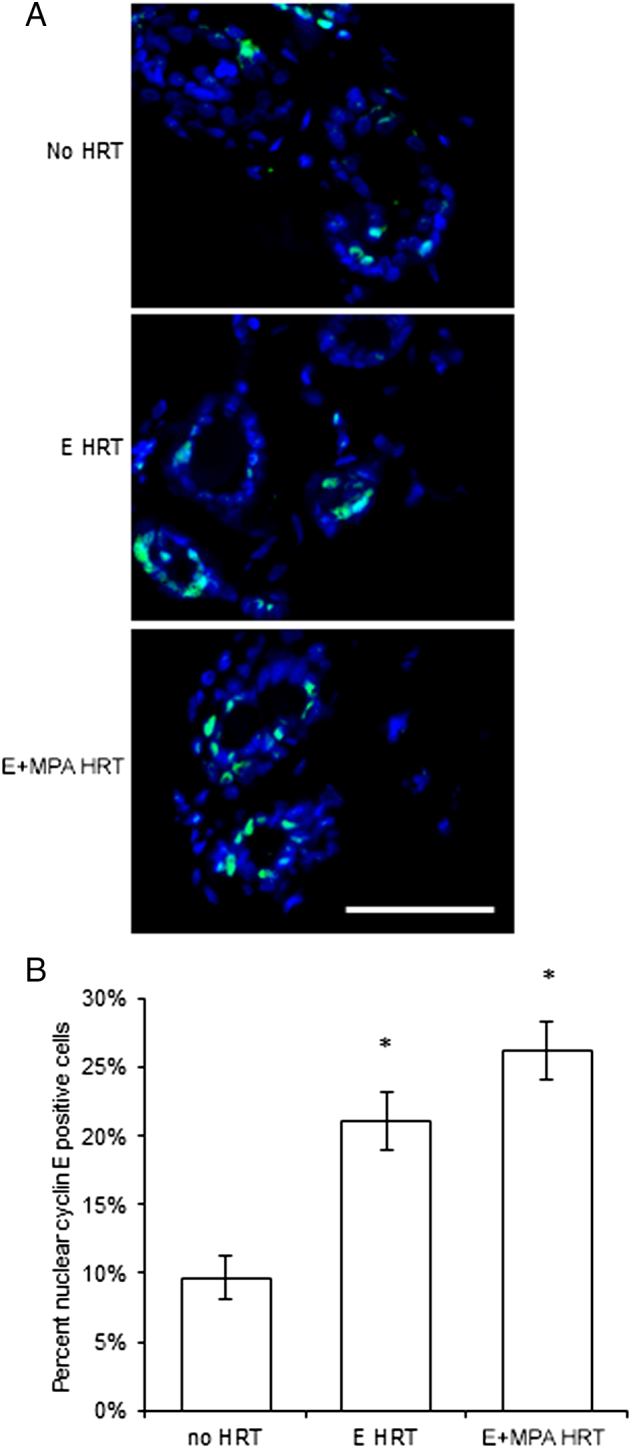
Nuclear cyclin E level was increased by E and E+MPA HRT. (A) Immunofluorescent detection of cyclin E (teal) within nuclei stained with DAPI (blue) in normal postmenopausal breast of women receiving no HRT, E HRT, or E+MPA HRT. Scale bar = 25 μm. (B) The percentage of nuclear cyclin E positive cells was increased in the postmenopausal breast by E and E+MPA HRT compared to no HRT (*P < .05). E+MPA HRT had a trend toward an increased percentage of nuclear cyclin E positive cells compared with E HRT (*P* = .11).

Nuclear localization of the cell cycle mediator, cyclin E, has been associated with cell cycle progression from G1-S phase [27], [28], so nuclear cyclin E was examined in postmenopausal breast epithelium as a potential mediator of proliferation (Figure 2). The percentage of nuclear cyclin E positive cells similarly significantly increased 2.2-fold with E alone and 2.7- fold with E+MPA HRT over no HRT. There was a trend toward increased cyclin E with E+MPA vs E alone (*P* = .11)

**Figure 3:**
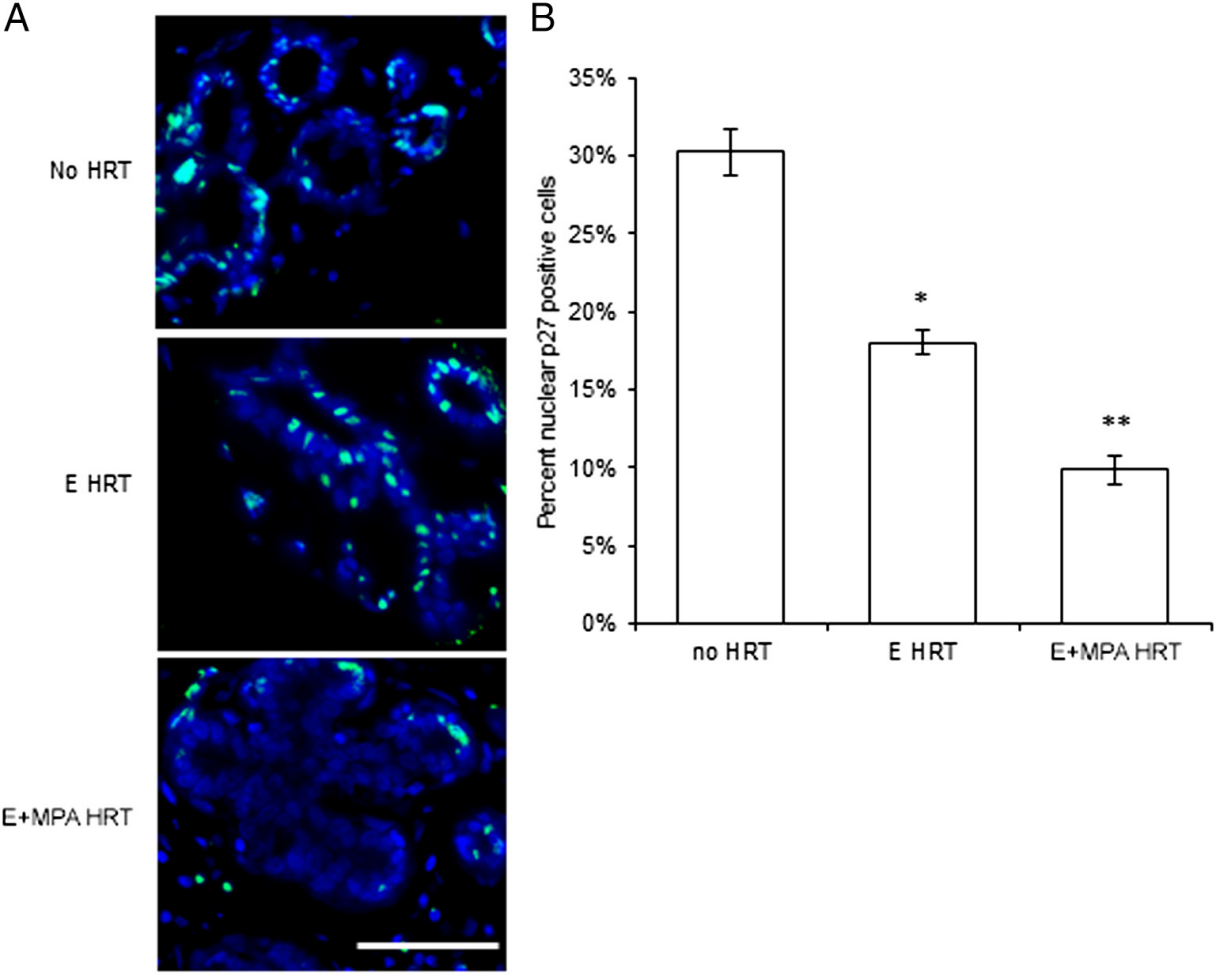
Decreased nuclear p27 level by E+MPA HRT. (A) Immunofluorescent detection of p27 (teal) within nuclei stained with DAPI (blue) in normal postmenopausal breast of women receiving no HRT, E HRT, or E+MPA HRT. Scale bar = 25 μm. (B) The percentage of nuclear p27 positive cells was decreased in the postmenopausal breast by E and E+MPA HRT compared to no HRT (*P < .05). E+MPA HRT further decreased the percentage of nuclear p27 positive cells compared with E HRT (**P < .05).

Nuclear localization of the cyclin-dependent kinase inhibitor p27 acts as a potent inhibitor of normal breast proliferation in the rat [29] and in human breast cancer [30]. Upon mitogenic stimulation, the cell cycle inhibitor p27 is rapidly degraded to allow cell cycle progression [31], [32]. Decreased levels of nuclear p27 were examined as a potential mediator of proliferation in response to HRT in the postmenopausal breast (Figure 3*A*). The percentage of nuclear p27 positive cells was decreased 1.6-fold by E-alone HRT and 3.1-fold by E+MPA compared with no HRT (Figure 3*B*). The decrease in the percentage of nuclear p27 positive cells by E+MPA HRT was significantly decreased beyond E-alone HRT.

**Figure 4:**
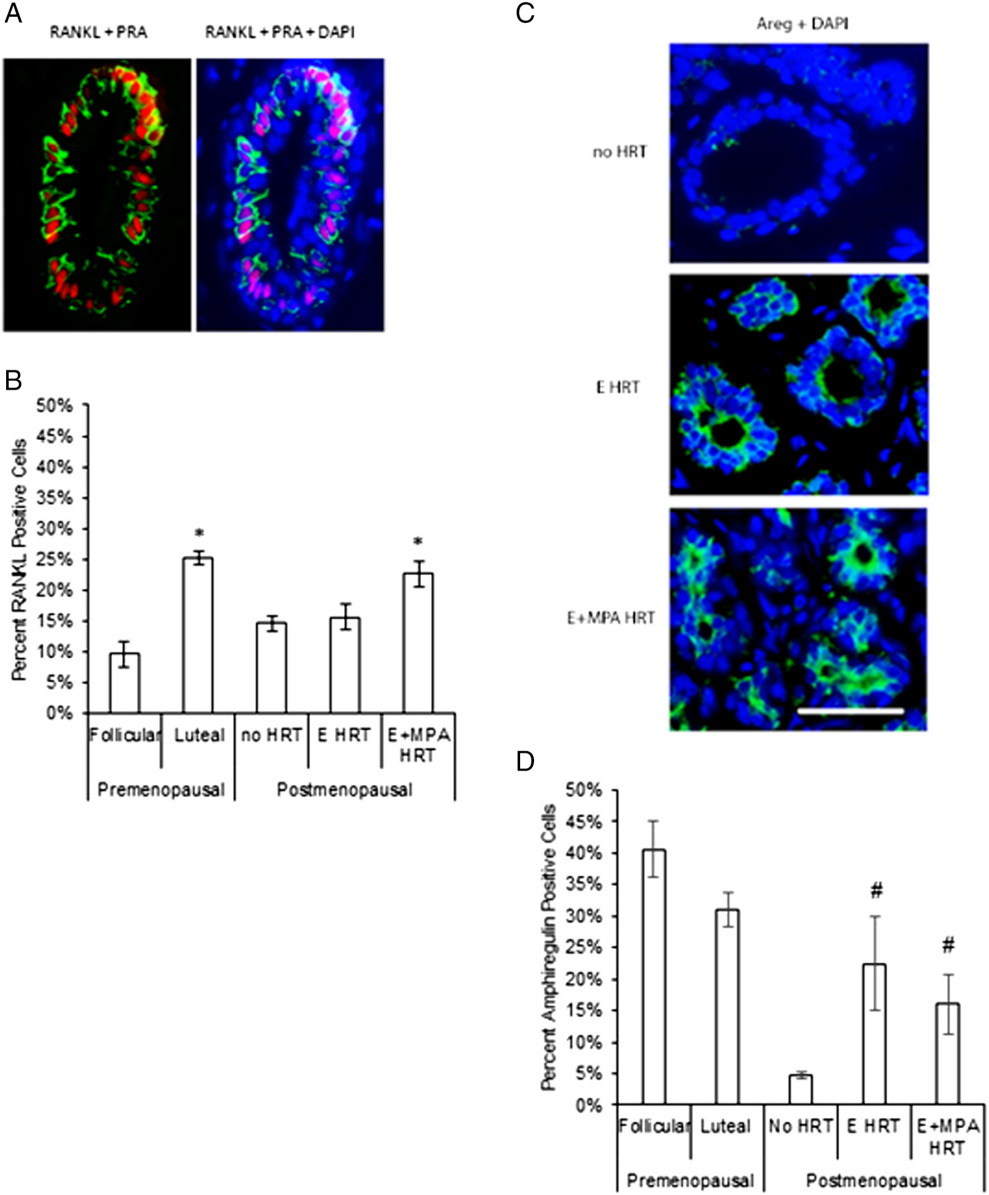
The effect of E vs E+MPA HRT on hormone-dependent paracrine factors RANKL and Amphiregulin. (A) Immunofluorescent detection of RANKL (green) and PRA (red) in normal postmenopausal breast of women receiving no HRT, E HRT, or E+MPA HRT and in the premenopausal breast. A representative image from E+MPA HRT is shown. Nuclei were stained with DAPI (blue). Scale bar = 25 μm. (B) The percentage of RANKL positive cells was increased in the postmenopausal breast by E+MPA HRT compared to no HRT (*P < .05). In premenopausal breast samples, the luteal phase of the cycle (n = 4), when progesterone is highest, had an increased percentage of nuclear p27 positive cells compared with the follicular phase (n = 3) (*P < .05). (C) Immunofluorescent detection of Amphiregulin (green) in normal postmenopausal breast of women receiving no HRT, E HRT, or E+MPA HRT. Nuclei were stained with DAPI (blue). Scale bar = 25 μm. (D) E and E+MPA HRT in the postmenopausal breast were associated with an increase in the percentage of Amphiregulin positive cells compared to no HRT (#*P* < .1). There was no significant difference between premenopausal samples in the luteal (n = 4) and follicular phases (n = 3).

RANKL is a specific mediator of P-induced proliferation in the human breast [33]. Increased levels of RANKL in the premenopausal human breast have been correlated with increased serum P levels, and expression of RANKL co-localized with PR expression [33]. RANKL expression in the postmenopausal breast co-localized with PRA expression (Figure 4*A*), and E+MPA HRT significantly increased the percentage of RANKL positive cells (Figure 4*B*). E HRT alone had no effect on RANKL levels. We also confirmed that the percentage of RANKL positive cells increased in the premenopausal breast during the luteal phase of the menstrual cycle, when serum P levels are highest. Thus, RANKL expression was similarly increased in the premenopausal breast in response to elevated P levels and in the postmenopausal breast with E+MPA.

Amphiregulin (Areg) also mediates P-induced proliferation in the mouse [34] and rat [35]. In addition, Areg can synergize with progestins to increase proliferation in breast cancer cells [35]. In the postmenopausal breast, both E-alone and E+MPA HRT produced similar small increases in the percentage of Areg positive cells compared to no HRT (Figure 4*C*).

### Proliferation was Similar in Estrogen vs. Estrogen + MPA HRT Breast Tumors

Differences in the proliferative response to E and E+MPA in the normal postmenopausal breast led us to examine whether those differences extended to breast cancers. Luminal breast tumors, expressing both ER and PR, were obtained from women who had taken E HRT or E+MPA HRT, and were used to examine PRA and PRB regulation, nuclear p27, RANKL, and the proliferation marker Ki67.

**Figure 5:**
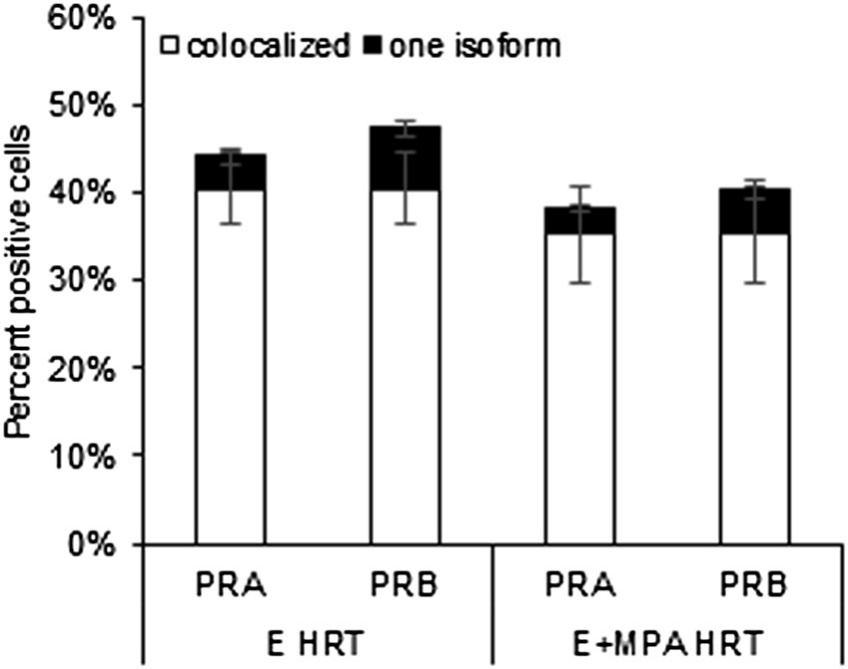
The percentage of PRA and PRB-expressing cells was increased in postmenopausal luminal breast tumors. Immunofluorescent detection of PRA and PRB in luminal breast tumors from women receiving either E HRT or E+MPA HRT revealed that the percentage of cells expressing PRA and PRB was increased in breast tumors compared to normal premenopausal breast or postmenopausal breast (*P* < .05, Figure 1). The percentage off cells expressing only PRA or only PRB was also increased compared to the normal premenopausal breast or postmenopausal breast (*P* < .05, Figure 1).

In the normal postmenopausal breast, PRA and PRB expression was maintained in the no HRT samples and increased by E or E+MPA HRT (Figure 1). The percentage of cells expressing PRA and PRB was increased 1.9- and 1.7-fold in E HRT and E+MPA luminal breast tumors, respectively (Figure 5), compared to no HRT normal postmenopausal breast (Figure 1), and were elevated compared to normal premenopausal breast levels (*P* < .05) (Figure 1). The percentage of PRA and PRB expressing cells in E and E+MPA HRT luminal breast tumors was also significantly increased compared to normal postmenopausal breast treated with E or E+MPA HRT. In both E and E+MPA HRT luminal breast tumors, the percentage of cells expressing only PRA or only PRB was also increased (*P* < .05) compared to the normal premenopausal breast and the postmenopausal breast with or without HRT (Figure 1, Figure 5).

**Figure 6:**
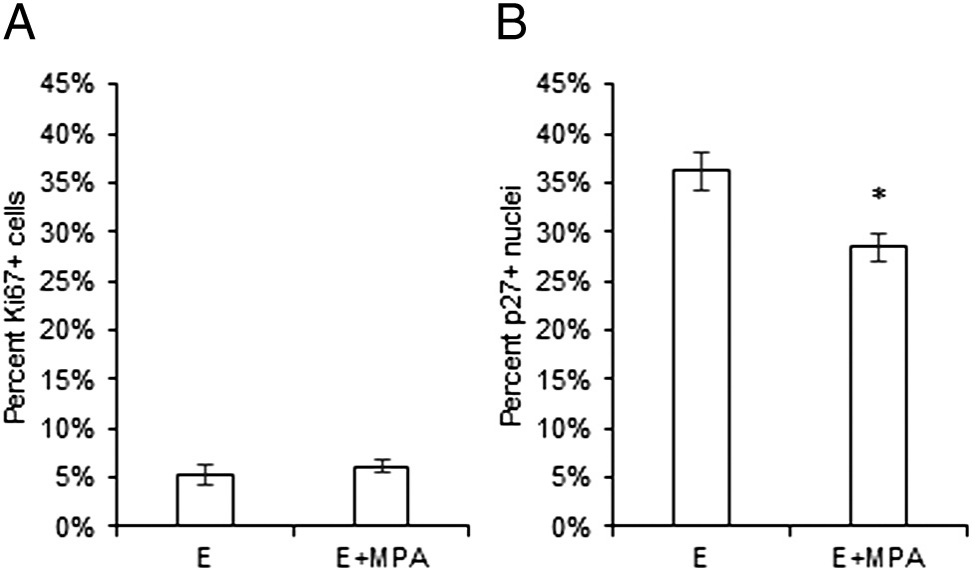
E+MPA HRT decreased nuclear p27 expression, but did not alter proliferation in breast tumors. (A) The percentage of cells expressing Ki67 in breast tumors was examined by immunofluorescence. No difference in Ki67 expression was detected between E and E+MPA HRT. (B) The percentage of cells expressing nuclear p27 was examined by immunofluorescence. Nuclear p27 expression was decreased by E+MPA HRT compared to E HRT (*P* < .05).

Immunofluorescent detection of the proliferation marker Ki67 in the E and E+MPA HRT luminal breast tumors showed no difference in proliferation (Figure 6*A*). There were no differences in RANKL expression in tumors between E and E+MPA HRT (data not shown). In contrast, the percentage of nuclear p27 expressing cells in tumors was significantly decreased by E+MPA HRT compared to E HRT (Figure 6*B*).

## Discussion

### PRA and PRB Regulation and Expression in Normal Breast and Breast Cancer

Similarly to the premenopausal breast [15], PRA and PRB co-expression in the same cells at similar levels was maintained in the normal postmenopausal human breast. PRA and PRB were predominantly expressed in luminal epithelial cells in the postmenopausal breast, which is similar to previous reports in the premenopausal breast [15]. Only PRB was occasionally expressed in myoepithelial cells in the postmenopausal breast, which was also similar to findings in the premenopausal breast [36], [37]. In contrast, mouse models have shown exclusive localization of PRA and PRB to luminal epithelial cells in mice, although not always to the same cells [38], and in rats PRB was frequently expressed in myoepithelial cells [29]. Thus, PRA and PRB co-localization is maintained in the postmenopausal breast and contrasts with mouse and rat models where PRA and PRB are less frequently co-localized.

E-dependent increases in overall PR expression have been shown in both the rat [35] and mouse models [39], but PRA and PRB isoform regulation in the postmenopausal human breast also differed from rat and mouse models. Co-localization of PRA and PRB expression with ERα expression in the postmenopausal breast suggests that E HRT increased PRA and PRB expression similarly through ERα. In contrast, PRA expression was E-dependent and PRB expression was E-independent in the rat [35], while PRA expression was E-independent and PRB expression required both E and P in the mouse [39]. In the postmenopausal breast, significant PRA and PRB expression was maintained, and both PRA and PRB expression increased in response to E HRT. Maintenance of PRA and PRB expression in the postmenopausal human breast may be E-independent, as in the mouse, or E-dependent, as in the rat, due to local aromatase activity producing enough E to maintain PRA and PRB expression. The lack of an MPA effect on PRA and PRB levels is consistent with the observations during the luteal phase of the menstrual cycle, when P levels are increased but there is no effect on PR isoform expression [15] and is also consistent with the number of PR+ cells remaining relatively constant throughout the menstrual cycle [40], [41].

While PRA and PRB were still often co-expressed in postmenopausal luminal breast tumors from women receiving E or E+MPA HRT, a higher percentage of cells expressed PRA and PRB in tumors than in the normal breast. This increase in PRA and PRB expression suggests either dysregulation of PRA and PRB expression or expansion of receptor positive cells in these tumors. Consistent with an alteration in the regulation of PRA and PRB expression during breast tumorigenesis [15], there was also an increase in the percentage of tumor cells expressing only PRA or only PRB. Alteration in the ratio of PRA to PRB has been associated with breast cancer progression and this alteration often increased the ratio toward PRA [14], which may be caused by increased turnover of active, ligand-bound PRB [17]. In the luminal tumors examined, there was no difference detected in PR isoform regulation between samples from women receiving E and E+MPA HRT. The lack of an impact of MPA on PRA and PRB ratios or level in tumors was similar to the normal postmenopausal breast, and suggests there was not increased PRB turnover in these tumors, in contrast to other tumor studies [17]. Future studies comparing luminal breast tumors from postmenopausal women who were not on HRT would provide a more complete analysis of the effect of E and E+MPA HRT on PRA and PRB regulation in breast tumors. In future studies, it may also be valuable to examine adjacent normal tissue paired with tumor samples to assess whether PR isoform ratios track between the two.

### The Effect of Estrogen versus Estrogen + MPA on Proliferation in the Normal Breast and Breast Cancer

In the current studies, nuclear cyclin E was slightly elevated and nuclear p27 was significantly decreased by E+MPA HRT compared to E alone, consistent with previous studies on these same samples where E+MPA HRT increased proliferation as measured by Ki67 and breast density compared to E alone [19]. Similar results were also obtained for premenopausal tissue in the previous study [19], where tissues from individuals in the luteal versus follicular phase of the menstrual cycle were compared. When localized to the nucleus, p27 can act as a potent inhibitor of proliferation in normal breast epithelium and in breast cancer [29], [30]. Rat mammary tumors developing in the presence of E+P exhibit increased proliferation associated with decreased nuclear p27 expression [42]. Progestin specifically regulated intracellular localization of p27 in human breast cancer cells [42] and progressive p27 loss has been observed during breast cancer progression [30], [43]. In the luminal breast cancers examined in these studies, nuclear p27 was decreased in patients who had received E+MPA HRT, but without any change in proliferation. Decreased nuclear p27, as in our E+MPA breast cancers, has been observed in primary breast tumors and linked with poor clinical outcome [44], [45]. Nuclear p27 also plays a role in differentiation [30] and cytoplasmic p27, which is poorly detectable by immunofluorescence, potentiates metastasis. Based on our findings in the normal postmenopausal breast, it is likely that p27 is involved in the increased proliferative response in the breast to MPA. In contrast, decreased nuclear p27 in breast tumors was not associated with increased proliferation, but may still influence tumor characteristics, such as aggressiveness. In light of a recent report that elevated nuclear p27 in normal breast epithelium is associated with reduced breast cancer risk [46], it would be valuable to examine adjacent normal tissue paired with tumor samples and compare the levels of nuclear p27 in the normal tissue of breast cancer patients to the normal tissue of those without breast cancer.

While nuclear cyclin E was increased by E+MPA HRT in the postmenopausal breast, it was also increased by E alone, suggesting that cyclin E is not specifically regulated by MPA, but is more generally associated with E-induced proliferation in the postmenopausal breast. Cyclin E is critical for the G1 to S transition and has been implicated in breast tumorigenesis (reviewed in [47]). Numerous cell cycle alterations have been associated with increased cyclin E: a decrease in G1-phase length, a more rapid transition from G1 to S phase, an increase in cyclin E kinase activity, and an increase in genomic instability [48], [49], [50], [51]. Although nuclear cyclin E increased significantly in the postmenopausal breast with E HRT, the addition of E alone HRT has not been associated with an increase in breast cancer risk [52], [53]. Thus, increased nuclear cyclin E is unlikely to play a direct role in increasing breast cancer risk with the addition of MPA to HRT.

RANKL was increased in the postmenopausal breast by E+MPA HRT, but not by E alone. In the mouse, RANKL is an essential mediator of P-induced proliferation [54] and influences expansion and regenerative potential of mammary stem cells [55], [56]. P regulates mouse mammary stem cells through a paracrine mechanism mediated by RANKL [33], [55], [56]. In the mouse [57], [58], premenopausal human [33], and postmenopausal primate [59], expression of RANKL within the breast was localized to PR-expressing cells. RANKL was also increased by E+MPA HRT in a postmenopausal primate model, but not with E alone [59]. In premenopausal breast tissue microstructures, RANKL was sufficient to induce breast proliferation and was required for P-induced proliferation [33]. Interestingly, RANKL not only acts within the normal mouse mammary gland, but can also influence progesterone-dependent mammary tumor formation [60], [61].

In the postmenopausal breast, both E and E+MPA HRT increased levels of Areg, an E [62] and P-induced paracrine factor [34] that promotes proliferation of mammary epithelial cells. However, our finding that Areg is similarly regulated by E and E+MPA HRT suggests that Areg expression is not likely to be associated with the increased risk of breast cancer with E+MPA HRT. These data taken together suggest that MPA acting through PRA/PRB increased RANKL expression in the postmenopausal breast and that RANKL-induced proliferation and influence on mammary stem cells may contribute to hormone-dependent tumor formation in the postmenopausal breast.

As noted in Results, 75% of the E-alone HRT subjects experienced a surgical menopause and their mean time on HRT was twice as long as that of women taking E+MPA HRT (15.8 vs. 7.4 y). In the latter group, only 7.7% experienced a surgical menopause; of those with no HRT, only 25% experienced a surgical menopause. This is a potentially confounding factor in the results. On the other hand, similar results between E and E+MPA HRT for Areg expression, while a differential response to E and E+MPA for RANKL expression, which is reported to correlate with serum levels of P [33], suggest that this is not the case.

### Progestins versus Progesterone in the Normal Breast

The effects of MPA in the postmenopausal breast were consistent with those of the luteal phase in the premenopausal breast, where increased natural P is associated with increased proliferation in the breast epithelium and increased breast lobule size and complexity [19], [24], [63], [64]. While the synthetic progestin MPA may bind to other members of the steroid receptor family, such as androgen receptor, glucocorticoid receptor, or mineralocorticoid receptor and it may have differential activity with the PR isoforms compared to P (reviewed in [65]), we consistently found similarities among all the downstream effects examined in the normal breast between the luteal phase in premenopausal women and E+MPA HRT in postmenopausal women. These results suggest that in the normal breast both MPA and P act through PRA and PRB to increase RANKL and decrease nuclear p27. Thus, there is consistent data both in the human and across mouse [39] and rat [29] models that E+P increases proliferation in the breast. Interestingly, the Postmenopausal Estrogen/Progestin Interventions trial has reported that hormone therapy with oral micronized P was associated with increased mammographic breast density [66], which is a risk factor for breast cancer. In contrast, observational studies using oral micronized P therapy have found that P did not increase breast cancer risk [67]. Taken together, the data suggest that P as part of any postmenopausal hormone therapy should be administered with caution and consideration of potential effects on the breast because the inclusion of P in HRT may lead to dysregulation of the p27 pathway as a checkpoint against enhanced proliferation.

The following are the supplementary data related to this article.

Supplemental Figure 1 Specific detection of human PRA and PRB. Specificity of anti-PRA and anti-PRB antibodies was examined using T47D breast cancer cells that express no PR (T47D-Y), PRA only (T47D-YA), PRB only (T47D-YB), or both PRA and PRB (T47D). (A) Immunofluorescence detection with rabbit polyclonal anti-PRB G1699 only detected PRB (green) in T47-YB cells. Immunofluorescent detection with mouse monoclonal anti-PRA hPRa7 only detected PRA (green) in T47-YA cells. Nuclei were counterstained with DAPI (blue). Scale bar = 25 μm. (B) Immunoblot for PRA and PRB. Rabbit polyclonal anti-PRB G1699 detected PRB in T47D-YB cells and T47D cells. Mouse monoclonal anti-PRB hPRa6 detected PRB in T47D-YB cells and T47D cells, although not as efficiently as G1699. Mouse monoclonal anti-PRA hPRa7 detected both PRA and PRB by immunoblot. (C) Rabbit polyclonal anti-PRB (G1699, green) and mouse monoclonal anti-PRB (hPRa6, red) showed similar patterns of PRB detection (merge, yellow). Scale bar = 25 μm. Supplemental Figure 1

Supplemental Figure 2 PRB co-localization with ERa and the myoepithelial cell marker p63. (A) Immunofluorescent detection of PRB (green) and ERa (red) in normal postmenopausal breast revealed co-localization of expression. Nuclei were counterstained with DAPI (blue). Scale bar = 25 μm. (B) (A) Immunofluorescent detection of PRB (green) and p63 (red), a marker of myoepithelial cells, showed that co-localization of expression occurred in less than 3% of PRB expressing cells in the postmenopausal breast across all treatment groups. Nuclei were counterstained with DAPI (blue). Scale bar = 25 μm. Supplemental Figure 2
