# Supplementary material for: The Proliferative Response to p27 Down-Regulation in Estrogen Plus Progestin Hormonal Therapy is Lost in Breast Tumors
Source: Transl Oncol. 2018 Mar 7;11(2):518–27. doi: 10.1016/j.tranon.2018.02.011 (PMC5884216; doi:10.1016/j.tranon.2018.02.011)
Supplement: Supplemental Figure 1 — Specific detection of human PRA and PRB. Specificity of anti-PRA and anti-PRB antibodies was examined using T47D breast cancer cells that express no PR (T47D-Y), PRA only (T47D-YA), PRB only (T47D-YB), or both PRA and PRB (T47D). (A) Immunofluorescence detection with rabbit polyclonal anti-PRB G1699 only detected PRB (green) in T47-YB cells. Immunofluorescent detection with mouse monoclonal anti-PRA hPRa7 only detected PRA (green) in T47-YA cells. Nuclei were counterstained with DAPI (blue). Scale bar = 25 μm. (B) Immunoblot for PRA and PRB. Rabbit polyclonal anti-PRB G1699 detected PRB in T47D-YB cells and T47D cells. Mouse monoclonal anti-PRB hPRa6 detected PRB in T47D-YB cells and T47D cells, although not as efficiently as G1699. Mouse monoclonal anti-PRA hPRa7 detected both PRA and PRB by immunoblot. (C) Rabbit polyclonal anti-PRB (G1699, green) and mouse monoclonal anti-PRB (hPRa6, red) showed similar patterns of PRB detection (merge, yellow). Scale bar = 25 μm. [file mmc1.pdf]

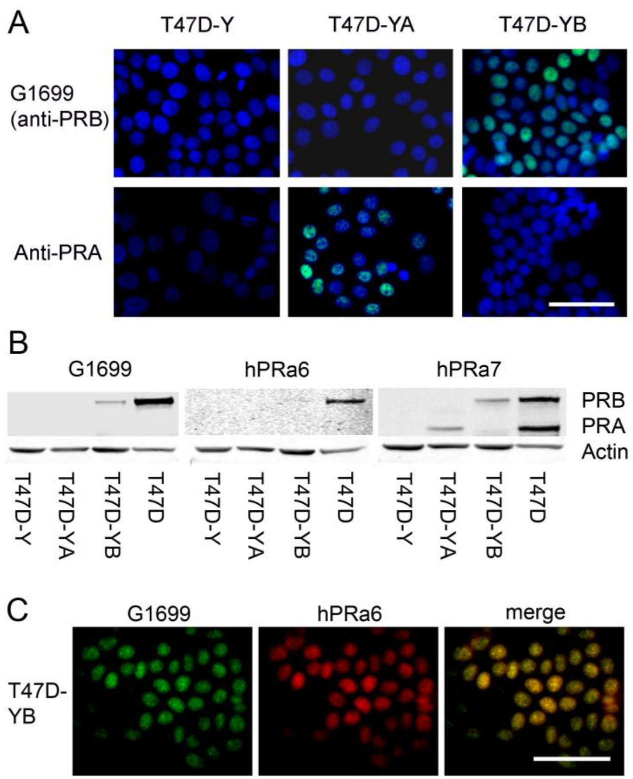

Supplemental Figure 1. Specific detection of human PRA and PRB. Specificity of anti-PRA and anti-PRB antibodies was examined using T47D breast cancer cells that express no PR (T47D-Y), PRA only (T47D-YA), PRB only (T47D-YB), or both PRA and PRB (T47D). (A) Immunofluorescence detection with rabbit polyclonal anti-PRB G1699 only detected PRB (green) in T47-YB cells. Immunofluorescent detection with mouse monoclonal anti-PRA hPRa7 only detected PRA (green) in T47-YA cells. Nuclei were counterstained with DAPI (blue). Scale bar = 25 μm. (B) Immunoblot for PRA and PRB. Rabbit polyclonal anti-PRB G1699 detected PRB in T47D-YB cells and T47D cells. Mouse monoclonal anti-PRB hPRa6 detected PRB in T47D-YB cells and T47D cells, although not as efficiently as G1699. Mouse monoclonal anti-PRA hPRa7 detected both PRA and PRB by immunoblot. (C) Rabbit polyclonal anti-PRB (G1699, green) and mouse monoclonal anti-PRB (hPRa6, red) showed similar patterns of PRB detection (merge, yellow). Scale bar = 25 μm.
