# Supplementary material for: The Proliferative Response to p27 Down-Regulation in Estrogen Plus Progestin Hormonal Therapy is Lost in Breast Tumors
Source: Transl Oncol. 2018 Mar 7;11(2):518–27. doi: 10.1016/j.tranon.2018.02.011 (PMC5884216; doi:10.1016/j.tranon.2018.02.011)
Supplement: Supplemental Figure 2 — PRB co-localization with ERa and the myoepithelial cell marker p63. (A) Immunofluorescent detection of PRB (green) and ERa (red) in normal postmenopausal breast revealed co-localization of expression. Nuclei were counterstained with DAPI (blue). Scale bar = 25 μm. (B) (A) Immunofluorescent detection of PRB (green) and p63 (red), a marker of myoepithelial cells, showed that co-localization of expression occurred in less than 3% of PRB expressing cells in the postmenopausal breast across all treatment groups. Nuclei were counterstained with DAPI (blue). Scale bar = 25 μm. [file mmc2.pdf]

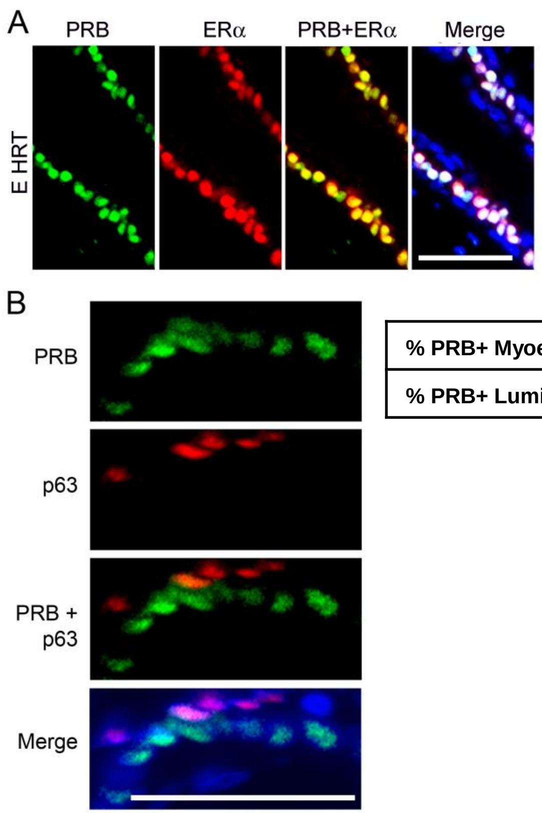

**% PRB+ Myoepithelial Cells**	**2.5**
**% PRB+ Luminal Epithelial Cells**	**97.5**

Supplemental Figure 2. PRB co-localization with ERa and the myoepithelial cell marker p63. (A) Immunofluorescent detection of PRB (green) and ERa (red) in normal postmenopausal breast revealed co-localization of expression. Nuclei were counterstained with DAPI (blue). Scale bar = 25 μm. (B) (A) Immunofluorescent detection of PRB (green) and p63 (red), a marker of myoepithelial cells, showed that co-localization of expression occurred in less than 3% of PRB expressing cells in the postmenopausal breast across all treatment groups. Nuclei were counterstained with DAPI (blue). Scale bar = 25 μm.
